# Effect of transarterial chemoembolization as postoperative adjuvant therapy for intermediate-stage hepatocellular carcinoma with microvascular invasion: a multicenter cohort study

**DOI:** 10.1097/JS9.0000000000000805

**Published:** 2023-10-06

**Authors:** Cailing Xiang, Xianbo Shen, Xinxin Zeng, Yuzhong Zhang, Zhongzhi Ma, Guocan Zhang, Xin Song, Tao Huang, Juan Yang

**Affiliations:** a Department II of General Surgery; b Department of Hepatobiliary Surgery, Hunan Provincial People’s Hospital (The First Affiliated Hospital of Hunan Normal University) Changsha; c Department of Hepatobiliary Surgery, The First Affiliated Hospital of Jishou University, Jishou, Hunan province; d Department of Minimally Invasive Intervention, Sun Yat-sen University Cancer Center; State Key Laboratory of Oncology in South China, Guangzhou, Guangdong province, People’s Republic of China

**Keywords:** hepatocellular carcinoma, liver resection, microvascular invasion, propensity score-matching, transarterial chemoembolization

## Abstract

**Background:**

Intermediate-stage hepatocellular carcinoma (HCC) with microvascular invasion (MVI) is associated with high recurrence rates and poor survival outcomes after surgery. This study aimed to evaluate the efficacy of postoperative transarterial chemoembolization (TACE) on prognosis of intermediate-stage HCC patients with MVI after curative liver resection (LR).

**Materials and methods:**

Patients who had intermediate-stage HCC with MVI and underwent curative LR between January 2013 and December 2019 at three institutions in China were identified for further analysis. Overall survival (OS) and recurrence-free survival (RFS) were compared between patients treated with and without postoperative TACE by propensity score-matching.

**Results:**

A total of 246 intermediate-stage HCC patients with MVI were enrolled, 137 entered into the LR group and 109 entered into the LR+TACE group. The 1-year, 3-year, and 5-year RFS rates were 42.0, 27.2, and 17.8% in LR+TACE group, and 31.8, 18.2, and 8.7% in LR group. The 1-year, 3-year, and 5-year OS rates were 81.7, 47.2, and 26.1% in the LR+TACE group, and 67.3, 35.6, and 18.5% in the LR group. Compared with LR alone, LR+TACE was associated with significantly better RFS [hazard ratio (HR), 1.443; 95% CI: 1.089–1.914; *P*=0.009] and OS (HR, 1.438; 95% CI: 1.049–1.972; *P*=0.023). No difference was observed with RFS and OS in single TACE and multiple TACE in the matched cohort.

**Conclusion:**

Postoperative adjuvant TACE could be beneficial for intermediate-stage HCC patients with MVI.

## Introduction

Highlights

- Postoperative transarterial chemoembolization (TACE) can significantly improve the prognosis of intermediate-stage hepatocellular carcinoma patients with microvascular invasion.
- A single TACE treatment after surgery can maximize the benefit without the need for multiple sessions.
- Postoperative TACE should be considered selectively in intermediate-stage hepatocellular carcinoma patients with microvascular invasion after curative liver resection.

Hepatocellular carcinoma (HCC) is one of the most common malignancies worldwide and the second leading cause of cancer-related mortality in China due to endemic chronic hepatitis B virus (HBV) infection^1,2^. Advances in diagnostic imaging and widespread application of screening programs in high-risk populations have allowed detection of HCC at an early-stage, but some patients still present with intermediate-stage HCC^3^. According to the Barcelona Clinic Liver Cancer (BCLC) algorithm for the treatment of HCC, intermediate-stage (stage B) HCC is defined as extensive multifocal disease confined to the liver without macrovascular invasion in patients with preserved liver function and the absence of cancer-related symptoms^4^. The prognosis of patients with intermediate-stage HCC varies differently with different treatments^5^. Currently, transarterial chemoembolization (TACE) is considered a first-line treatment for patients with intermediate-stage HCC^4^. However, European and Japanese guidelines for the management of HCC indicate that not all patients with intermediate-stage HCC are eligible for TACE, nor do all benefit from this treatment^6,7^. Thus, there is a need for other first-line treatment options for patients with intermediate-stage HCC.

Typically, liver resection (LR) is only favorable for the treatment of patients with early‑stage HCC who have good liver function^4,6^. With the improvement of surgical technique, LR has proven to be an effective option for certain patients with intermediate-stage HCC^8,9^. Some have even reported that in HCC patients with preserved liver function, the presence of large or multinodular tumors, macrovascular invasion, and/or portal hypertension should not be contraindications for LR^10^. However, the long-term survival rate of patients with intermediate-stage HCC after curative LR is still unsatisfactory due to the high incidence of tumor recurrence. Higher rates of tumor recurrence after curative LR usually lead to reduced overall survival (OS) in patients with intermediate-stage HCC, particularly those with microvascular invasion (MVI)^11^. MVI refers to the presence of micrometastatic HCC emboli within the vessels of the liver as seen on surgical histopathology^12^, and it is a major risk factor for poor prognosis in patients with intermediate-stage HCC after LR^13^. Therefore, it is of great significance to find effective therapies for such patients.

Some studies indicated that adjuvant radiotherapy^14^, sorafenib^15^, might decrease tumor recurrence after curative resection of HCC with MVI. However, none of these approaches have been recommended as universally accepted adjuvant therapy by current scientific guidelines after curative resection^6,16^. Currently, the main adjuvant therapy for the prevention of postoperative recurrence in patients with early-stage HCC with MVI is adjuvant TACE^17–19^. However, whether the postoperative adjuvant TACE could reduce recurrence and prolong the survival time of patients with MVI is still controversial. In addition, with more studies of MVI in recent years, the application of postoperative TACE in the adjuvant therapy of patients with early-stage HCC with MVI after curative LR has attracted much attention, but whether it can effectively reduce the recurrence of patients with intermediate-stage HCC with MVI and prolong the survival time is still unclear. In this multicenter study, we aimed to evaluate the effectiveness of postoperative TACE on the prognosis of patients with intermediate-stage HCC with MVI after curative LR.

## Methods

### Patients and study design

We retrospectively reviewed data on consecutive patients diagnosed with intermediate-stage HCC (BCLC stage B) who had undergone curative LR and postoperative TACE from January 2013 to December 2019 at three hospitals. The study protocol was consistent with the ethical guidelines of the 1975 Declaration of Helsinki and was approved by the Institutional Review Board of each participating institution. This study was reported in line with the strengthening the reporting of cohort, cross-sectional, and case–control studies in surgery (STROCSS) criteria^20^ (Supplemental Digital Content 1, http://links.lww.com/JS9/B175).

**Figure 1:**
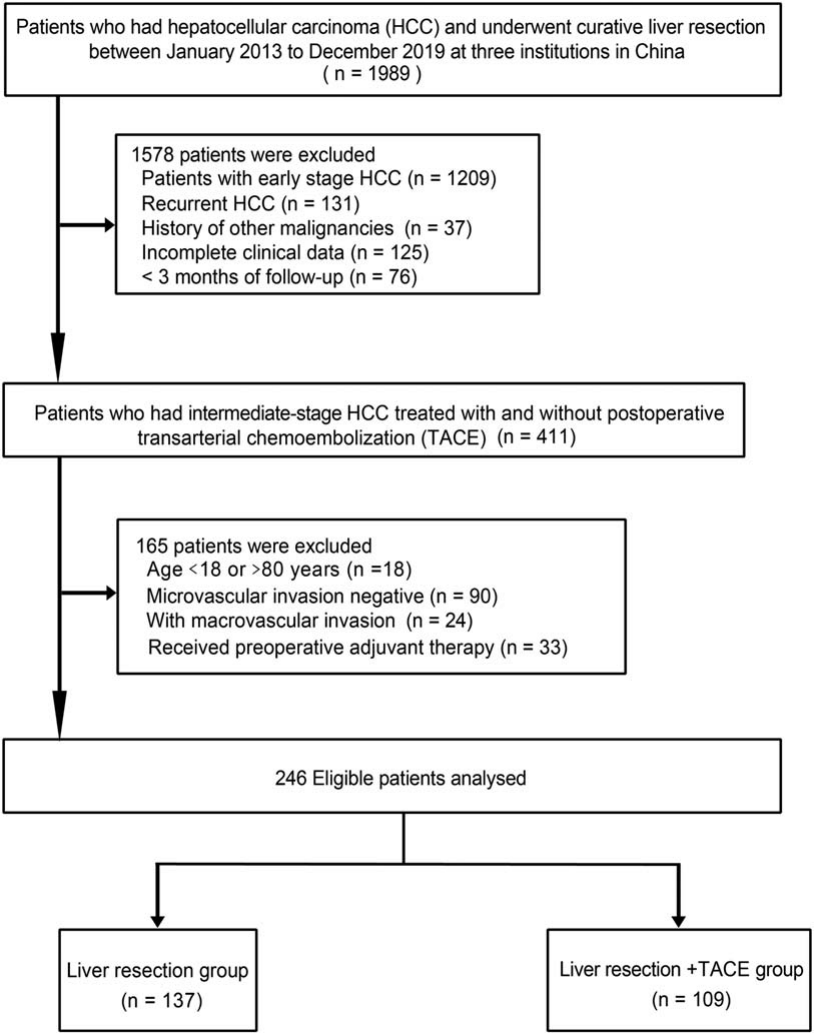
Flowchart of patient selection.

Patients who met the following criteria were enrolled: (1) age 18–80 years; (2) patients with intermediate-stage HCC (BCLC stage B) with MVI, where MVI was diagnosed by postoperative pathology; (3) curative LR (complete resection of the tumor without any tumor tissue at the cutting edge^21^); (4) no macrovascular invasion; (5) without any preoperative anticancer treatments; (6) Child-Pugh class A or B; (7) no history of other malignancies; patients were excluded from the analysis for any of the following reasons: (1) HCC patients with MVI negative; (2) recurrent HCC; (3) received preoperative adjuvant therapy; (4) early-stage HCC (BCLC stage 0-A); (5) incomplete clinical data; (6) lost to follow-up within 3 months after treatment. A total of 246 consecutive patients who were treated with either LR alone (LR group, *n*=137) or LR plus adjuvant TACE (LR+TACE group, *n*=109) were included in the analysis as the study population. The flowchart of patient selection is presented in Figure 1.

### LR and postoperative adjuvant TACE

LR was performed by experienced surgeons. Surgical plan was based on tumor number, tumor size, tumor location, and liver function. The hepatectomy method contains nonanatomical resection and anatomical resection, and the extent was defined using the Brisbane 2000 Terminology of Liver Anatomy and Resections^22^. LR procedures have been described in detail in our previous study^23^. Curative LR was defined as the complete removal of all detected tumors without involving any major branch of the portal or hepatic veins, without invasion of adjacent organs and without lymph node or distant metastasis, and tumor-free margins confirmed by histopathology.

After 4–6 weeks of hepatectomy, when the liver function of the patient with MVI had recovered, TACE was performed for the entire remnant liver. Whether patients followed the adjuvant TACE or not depended on their treatment compliance, economic status, or other social factors. TACE was conducted using the Seldinger technique, and hepatic angiography, computed tomography (CT) angiography, or both were performed to detect any obvious tumor stains in the remnant liver. Hepatic artery angiography was performed using a 5 Fr (RH or Yashiro) catheter. The embolization emulsion was a mixture of Epirubicin (Farmorubicin; Pharmacia) 30 mg–60 mg, Lobaplatin (Chang’an International Pharmaceutical) 30 mg–50 mg, and Lipiodol (Laboratorie Guerbet) 5 ml–10 ml. The doses of the agents contained in the embolization emulsion were selected based on patient age, weight, comorbidity, and anticipated tolerance. The decision about the number of TACE was based on the presence of a poorly differentiated tumor on surgical pathology, narrower resection margins, or on abnormal postoperative alpha-fetoprotein (AFP) levels. Repeat whole liver TACE is not recommended for patients who do not recover after liver function impairment. After 1 month of follow-up evaluation, a CT scan was performed to determine the effects of TACE.

### Outcomes and definitions

The primary endpoint for the study was OS, defined as the time from the date of curative resection to the date when the patients died or the last follow-up. The secondary endpoints included recurrence-free survival (RFS) and efficacy. RFS is defined as the time from the date of curative resection to the date at which HCC recurred. The diagnosis of HCC was confirmed according to histopathological evidence after LR. The BCLC staging system was used to evaluate the HCC stage^4^. The histologic grade of tumor differentiation was evaluated using the Edmondson-Steiner (ES) classification^24^. Cirrhosis was defined histologically by the findings of resected liver specimens. We used the Albumin-Bilirubin (ALBI) grade to evaluate liver function^25^.

### Follow-up

The follow-up protocol and management of recurrent HCC have been described in our previous study^23^. All patients with MVI were first evaluated for recurrence 4 weeks after LR by contrast-enhanced CT or MRI and a measurement of the serum AFP level. The patients were then re-evaluated every 3 months by measurement of their serum AFP level and by contrast-enhanced CT or MRI until death or dropout from the follow-up program. This investigation’s follow-up period concluded on 30 March 2023.

### Statistical analysis

Propensity score-matching analysis was involved to minimize alternative factors and sampling bias between the two groups. Propensity scores were computed using the following 14 variables: sex, age, tumor number, maximum tumor diameter, tumor differentiation, capsule, blood loss, blood transfusion, resection pattern, resection margin, aspartate aminotransferase (AST), alanine aminotransferase (ALT), AFP, ALBI grade. For PSM, a nearest-neighbor 1:1 matching scheme with a caliper size of 0.2 was used (Supplementary Figure 1, Supplemental Digital Content 2, http://links.lww.com/JS9/B176). OS and RFS were compared between the LR group and LR+TACE group in a propensity score-matched cohort by a log-rank test.

The comparison of continuous variables with or without normal distribution was analyzed with the Student *t*-test and the Wilcoxon rank test, respectively. *χ*^2^ and Fisher’s test were applicated for the analysis of categorical variables. OS and RFS rates were calculated using the Kaplan–Meier method and compared using the log-rank test. Multivariate analyses were performed by the Cox proportional hazards regression model with a stepwise selection of variables. The statistical analyses were performed using the Statistical Package for the Social Science (SPSS) software (version 22.0, SPSS Inc.) for Windows and R software for Windows (Version 4.1.3; http://www.r-project.org). *P* <0.05 was considered significant.

## Results

### Baseline characteristics

This study recruited 246 patients with a median age of 51.0 (range: 18–80) years. Among them, 217 (88.2%) patients were male. 137 (55.7%) patients received curative LR alone (LR group); 109 (44.3%) patients received curative LR and postoperative TACE (LR+TACE group). A total of 209 (85.0%) patients were HBsAg-positive. In the entire cohort, compared with the LR group, the LR+TACE group had significantly more patients with blood transfusion (27.5 vs. 16.8%; *P*=0.042), median intraoperative blood loss (400 ml vs. 300 ml; *P*<0.001), nonanatomical LR (83.5 vs. 70.8%; *P*=0.020) and resection margins of 0.5 cm or less (71.6 vs. 54.0%; *P*=0.005). PSM (1:1 matching) analysis generated two new cohorts of 90 and 90 patients in the LR group and LR+TACE group, respectively, and the characteristics of the two groups were balanced, with the standardized mean difference less than 10% for all baseline variables (Supplementary Figure 2, Supplemental Digital Content 2, http://links.lww.com/JS9/B176). The clinicopathological data of patients in the entire cohort and in the matched cohort are summarized in Table 1.

**Table 1:** Baseline characteristics of intermediate-stage hepatocellular carcinoma patients with microvascular invasion in different treatment groups.

	Entire cohort			Propensity score-matched cohort (1:1 ratio)		
Characteristics	LR group (*n*=137)	LR + TACE group (*n*=109)	*P*	LR group (*n*=90)	LR + TACE group (*n*=90)	*P*
Age^a^, years	52 [19–80]	51 [18–79]	0.353	53 [19–80]	53 [18–79]	0.503
Sex male, n (%)	123 (89.8)	94 (86.2)	0.629	77 (85.5)	79 (87.8)	0.661
Hemoglobin^a^, g/l	146 [100–195]	142 [90–210]	0.351	143 [100–195]	142 [90–210]	0.628
Platelet^a^, 10^9^/l	173 [70–485]	171 [67–450]	0.371	172 [70–485]	172 [70–450]	0.626
ALT^a^, U/l	37.5 [9.8–281.5]	38.5 [8.3–310.8]	0.319	37.8 [9.8–281.5]	38.0 [8.3–292.1]	0.528
CRE^a^, μmol/l	72.4 [34.7–232.5]	70.0 [21.9–195.0]	0.810	71.6 [34.7–232.5]	70.0 [21.9–195.0]	0.645
PT^a^, s	11.9 [10.1–14.7]	12.0 [10.1–14.8]	0.882	11.9 [10.1–14.7]	11.9 [10.1–14.8]	0.376
HBsAg-positive, *n* (%)	116 (84.7)	93 (85.3)	0.887	76 (84.4)	77 (85.5)	0.835
Cirrhosis yes, *n* (%)	90 (66.0)	76 (69.7)	0.503	64 (71.1)	65 (72.2)	0.869
Tumor diameter^a^, cm	7.0 [4.9–18.0]	7.3 [5.0–19.3]	0.153	7.0 [4.9–18.0]	7.0 [5.0–19.3]	0.395
Blood loss^a^, ml	300 [100–1700]	400 [100–2000]	<0.001	400 [200–1700]	400 [100–2000]	0.201
ALBI grade, *n* (%)			0.302			0.466
Grade 1	55 (40.1)	49 (45.0)		39 (43.3)	37 (41.1)	
Grade 2	81 (59.1)	57 (52.3)		50 (55.5)	53 (58.9)	
Grade 3	1 (0.8)	3 (2.8)		1 (1.2)	0 (0.0)	
AFP, *n* (%)			0.877			0.651
≤400 ng/ml	73 (53.3)	57 (52.3)		50 (55.6)	53 (58.9)	
>400 ng/ml	64 (46.7)	52 (47.7)		40 (44.4)	37 (41.1)	
Capsule, *n* (%)			0.981			0.824
Complete	35 (25.5)	29 (26.6)		23 (25.6)	25 (27.7)	
Incomplete	43 (31.4)	34 (31.2)		32 (35.6)	34 (37.8)	
Noncapsule	59 (43.1)	46 (42.2)		35 (38.8)	31 (34.5)	
Differentiation, *n* (%)			0.177			0.305
I–II	95 (69.3)	84 (77.1)		70 (77.8)	64 (71.1)	
III–IV	42 (30.7)	25 (22.9)		20 (22.2)	26 (28.9)	
Tumor number, *n* (%)			0.889			0.856
≤3	102 (74.5)	82 (75.2)		70 (77.8)	71 (78.9)	
>3	35 (25.5)	27 (24.8)		20 (22.2)	19 (21.1)	
Resection pattern, *n* (%)			0.020			1.000
Anatomic	40 (29.2)	18 (16.5)		16 (17.8)	16 (17.8)	
Nonanatomic	97 (70.8)	91 (83.5)		74 (82.2)	74 (82.2)	
Resection margin, *n* (%)			0.005			0.948
≤0.5 cm	74 (54.0)	78 (71.6)		58 (64.4)	60 (66.7)	
0.5–1.0 cm	33 (24.1)	22 (20.2)		21 (23.4)	20 (22.2)	
>1.0 cm	30 (21.9)	9 (8.2)		11 (12.2)	10 (11.1)	
Blood transfusion, *n* (%)			0.042			0.128
No	114 (83.2)	79 (72.5)		77 (85.6)	69 (76.7)	
Yes	23 (16.8)	30 (27.5)		13 (14.4)	21 (23.3)	

Data are *n* (%) and ranges.

a Presented as median and ranges.

AFP, alpha-fetoprotein; ALBI, albumin-bilirubin; ALT, alanine aminotransferase; CRE, creatinine; HBsAg, hepatitis B surface antigen; LR, liver resection; PT, prothrombin time; TACE, transarterial chemoembolization.

### Impact of postoperative TACE on the OS of intermediate-stage HCC patients with MVI

**Figure 2:**
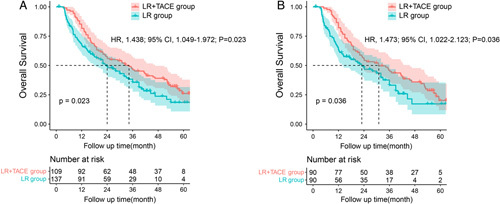
Kaplan–Meier analysis of overall survival (OS) in the entire cohort (2A) and in the propensity score-matched cohort (2B) of intermediate-stage hepatocellular carcinoma (HCC) patients with microvascular invasion (MVI) after curative liver resection.

During the follow- up period, 162 (65.9%) patients died in the entire cohort. Of the patients who had died, 88 (64.2%) patients in the LR group and 74 (67.9%) patients in the LR+TACE group, respectively. Patients in the entire cohort, the 1-year, 3-year, and 5-year OS rates were 81.7, 47.2, and 26.1% in LR+TACE group, and 67.3, 35.6, and 18.5% in LR group, respectively (Fig. 2A). After propensity matching, the 1-year, 3-year, and 5-year OS rates were 82.2, 46.0, and 20.2% in LR+TACE group, and 63.4, 33.1, and 17.2% in LR group, respectively (Fig. 2B). The LR+TACE group had significantly better OS than the LR group both in the entire cohort [hazard ratio (HR), 1.438; 95% CI: 1.049–1.972; *P*=0.023) and in the matched cohort (HR, 1.473; 95% CI: 1.022–2.123; *P*=0.036).

Univariate analysis of RFS and OS were presented in Supplementary Table 1 (Supplemental Digital Content 2, http://links.lww.com/JS9/B176). The multivariate Cox regression analysis was performed in the entire cohort and the results revealed that treatment pattern, tumor number, tumor differentiation, cirrhosis, and ALBI grade were significant factors associated with OS in patient with intermediate-stage HCC with MVI (Table 2).

**Table 2:** Multivariable analysis of prognostic factors for recurrence-free survival (RFS) and overall survival (OS) in intermediate-stage hepatocellular carcinoma (HCC) patients with microvascular invasion (MVI) after curative liver resection.

	Recurrence-free survival		Overall survival	
Variables	Hazard ratio (95% CI)	*P*	Hazard ratio (95% CI)	*P*
Treatment pattern				
LR+TACE	Reference	<0.001	Reference	<0.001
LR	1.98 (1.54–2.55)		1.73 (1.28–2.34)	
Tumor number				
≤3			Reference	
>3			2.22 (1.58–3.14)	<0.001
Differentiation				
I–II	Reference		Reference	
III–IV	1.68 (1.29–2.19)	<0.001	1.58 (1.15–2.15)	0.004
Maximum tumor size, cm				
>3, ≤5	Reference			
5–10	1.49 (1.01–2.22)	0.048		
>10	2.21 (1.39–3.50)	0.001		
Cirrhosis				
No	Reference		Reference	
Yes	1.84 (1.43–2.36)	<0.001	1.97 (1.45–2.69)	<0.001
ALBI grade				
Grade 1	Reference		Reference	
Grade 2	1.50 (1.18–1.92)	0.001	1.51 (1.11–2.06)	0.009
Grade 3	4.48 (1.71–9.56)	<0.001	3.91 (1.51–8.09)	0.005

ALBI, albumin-bilirubin; LR, liver resection; TACE, transarterial chemoembolization.

### Impact of postoperative TACE on the RFS of intermediate-stage HCC patients with MVI

**Figure 3:**
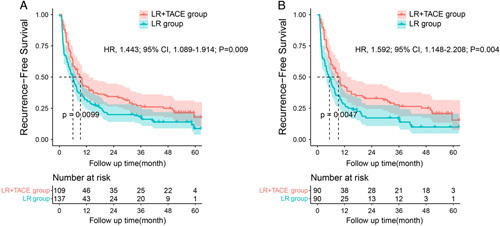
Kaplan–Meier analysis of recurrence-free survival (RFS) in the entire cohort (3A) and in the propensity score-matched cohort (3B) of intermediate-stage hepatocellular carcinoma (HCC) patients with microvascular invasion (MVI) after curative liver resection.

Patients had developed tumor recurrence in a total of 200 (81.3%) patients during follow- up with 116 (84.7%) patients in the LR group and 84 (77.1%) patients in the LR+TACE group in the entire cohort. Before propensity matching, the 1-year, 3-year, and 5-year RFS rates were 42.0, 27.2, and 17.8% in LR+TACE group, and 31.8, 18.2, and 8.7% in LR group, respectively (Fig. 3A). After propensity matching, the 1-year, 3-year, and 5-year RFS rates were 41.9, 27.8, and 15.5% in LR+TACE group, and 28.4, 17.3, and 10.0% in LR group, respectively (Fig. 3B). The LR+TACE group had significantly better RFS than the LR group both in the entire cohort (HR, 1.443; 95% CI: 1.089–1.914; *P*=0.009) and in the matched cohort (HR, 1.592; 95% CI: 1.148–2.208; *P*=0.004).

The multivariate Cox regression analysis was performed in the entire cohort and the results revealed that treatment pattern, tumor differentiation, maximum tumor size, cirrhosis, and ALBI grade were significant factors associated with tumor recurrence in patient with intermediate-stage HCC with MVI (Table 2).

### Efficacy of the number of postoperative TACE treatments on patient prognosis

**Figure 4:**
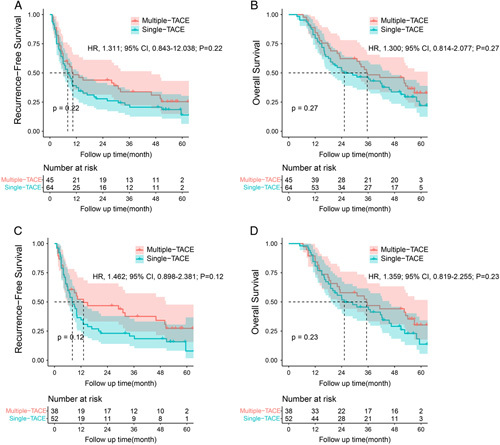
Kaplan–Meier analysis of recurrence-free survival (RFS) in the entire cohort (4A) and in the propensity score-matched cohort (4C), and overall survival in the entire cohort (4B) and in the propensity score-matched cohort (4D) of intermediate-stage hepatocellular carcinoma (HCC) patients with microvascular invasion (MVI) who were treated with either single transarterial chemoembolization (TACE) or multiple TACE after curative liver resection.

In order to judge the effect of the number of postoperative TACE treatments on the prognosis of intermediate-stage HCC patients with MVI, we further analyzed the frequency and efficacy of postoperative TACE treatment in the LR+TACE group. Patients were classified into two groups: (a) patients received only one postoperative adjuvant TACE before the tumor recurrence (single TACE group); (b) patients received two or more postoperative adjuvant TACE before the tumor recurrence (multiple TACE group). No difference was observed with RFS (Fig. 4A; HR, 1.311; 95% CI: 0.843–12.038; *P*=0.22) and OS (Fig. 4B; HR, 1.300; 95% CI: 0.814–2.077; *P*=0.27) in single TACE group and multiple TACE group in the entire cohort. After propensity matching, the medium RFS and OS were 8.0 months, 13.1 months, and 25.2 months, 35.3 months in single TACE group and multiple TACE group, respectively. Similarly, no difference was observed with RFS (Fig. 4C; HR, 1.462; 95% CI: 0.898–2.381; *P*=0.12) and OS (Fig. 4D; HR, 1.359; 95% CI: 0.819–2.255; *P*=0.23) in single TACE group and multiple TACE group in the matched cohort.

## Discussion

In the present study, we evaluated the impacts of postoperative adjuvant TACE on the prognosis of patients who underwent curative LR for intermediate-stage HCC with MVI. Results from this study suggest that postoperative TACE significantly improved the RFS and OS of intermediate-stage HCC patients with MVI compared with those who received LR alone, and postoperative adjuvant TACE was found to be an independent risk factor of RFS and OS. In addition, no difference was observed between postoperative single TACE and multiple TACE in improving the prognosis of patients with intermediate-stage HCC with MVI.

Currently, the BCLC staging system recommends TACE as the first-line treatment for intermediate-stage HCC^4^. However, some investigators have argued that LR should be considered as a first-line treatment for intermediate-stage HCC, given that the evidence supporting TACE as a first-line treatment may not be strong enough, and that the outcomes with LR may be better^26^. Furthermore, a clinical randomized controlled trial showed that LR is more aggressive than TACE and should be considered as a first-line treatment for intermediate-stage HCC^27^. Additionally, studies in Asian patients with intermediate-stage HCC have demonstrated that even the large number of HCC tumors should not be a contraindication to treatment with LR^28–30^. Of course, other tumor-related and liver-related factors need to be considered when determining the appropriate treatment for intermediate-stage HCC. Our results are consistent with those of the previous studies. For patients with intermediate-stage HCC who have good liver function and appear to be candidates for a curative resection, LR should be prioritized as a first-line treatment.

Although advances in surgical techniques, perioperative care, and accurate patient selection have gradually reduced surgical complications and mortality in recent years, the substantial recurrence rate after LR for intermediate-stage HCC remains a challenge and has led to efforts to find other effective adjuvant therapies^31^. Postoperative TACE has been considered one of those options. However, whether the postoperative TACE could reduce recurrence and prolong the survival time of HCC patients is still controversial. At the same time, most of the studies on postoperative TACE focus on early-stage HCC, while the studies on intermediate-stage HCC are still lacking. In some recent studies, postoperative TACE failed to demonstrate efficacy in patients with HCC^32,33^. In contrast, others have reported that postoperative TACE may achieve higher OS and RFS rates than surgical resection alone, as well as that TACE can be used as an effective adjuvant treatment after LR^17–19^. In our study, the result showed that postoperative TACE significantly improved the RFS and OS of intermediate-stage HCC patients with MVI in both the entire and propensity-matched cohorts, suggesting that for patients with MVI-positive intermediate-stage HCC, the addition of TACE after LR may be preferable. Moreover, postoperative TACE was also identified as an independent factor affecting both their RFS and OS.

The presence of MVI in HCC, usually discovered on surgical pathology, is a major obstacle to the achievement of good long-term survival rates after LR for patients^12,34^. It correlates with histologic grade, tumor diameter, and number of nodules^12^. MVI is present in 30–60% of nodules 2–5 cm in size, and up to 60–90% of tumors larger than 5 cm^35^, and it has been recognized as one of the most powerful predictors of tumor recurrence in patients with HCC after curative hepatectomy^11,13,23^. However, since MVI cannot be recognized preoperatively, it can only be accurately diagnosed by postoperative histopathology. Therefore, to decrease tumor recurrence, postoperative adjuvant therapy is essential for HCC patients with MVI. As the main adjuvant therapy after curative hepatectomy, TACE may play an important role in preventing tumor recurrence in HCC patients with MVI. Two recent interesting studies have shown that postoperative TACE significantly improved the RFS and OS of HCC patients with MVI^17,19^. It must be noted that most (more than 71% in Wang *et al*., and more than 65% in Sun *et al*.) of the patients in their study were early-stage HCC patients. However, there is still a lack of sufficient evidence for patients with intermediate-stage HCC. Our study confirms that postoperative TACE is equally helpful for intermediate-stage HCC patients with MVI. Thus, the findings of our study complement those published by Sun *et al*.

Currently, although TACE has been proven to be an effective strategy for the prevention of recurrence in HCC patients with high-risk factors^17,19,36^, the timing and times of TACE as an adjuvant therapy remained indistinct. Tong *et al*.^37^ reported that a single postoperative TACE treatment was beneficial for patients with HCC and repeated TACE did not increase patient benefit, but their study focused on patients in American Joint Committee on Cancer (AJCC) stage I and tumors less than 5 cm in size. Feng *et al*.^38^ reported that patients who received multiple TACE treatments after curative hepatectomy had better RFS and OS rates than patients who had a single TACE treatment. However, their study was limited to patients with HCC that met the Milan criteria, including no tumor larger than 5 cm. In the present study, for intermediate-stage HCC patients with MVI, no difference was observed with RFS and OS in single TACE group and multiple TACE group both in the entire and matched cohorts. In theory, compared to a single TACE treatment, multiple TACE after LR should have improved the outcomes of patients with intermediate-stage HCC, by further eradicating residual any occult intrahepatic satellite lesions not identified by imaging^39^. However, patients with intermediate-stage HCC often have a large tumor burden as well as impaired liver function and immune systems^40^. In addition, TACE may accelerate the deterioration of liver function, contribute to the suppression of host immunity against tumor progression, and negatively impact the regeneration of hepatocytes^41,42^. Hence, patients with intermediate-stage HCC had undergone surgical removal of a large volume of liver tissue, and balanced against these potentially negative sequelae, it is possible that those who received multiple TACE treatments obtained relatively limited benefit from more than a single TACE treatment, ultimately resulting in outcomes that were not better. In any case, our findings suggest that for patients with intermediate-stage HCC, a single TACE treatment after surgery can maximize the patient benefit without the need for multiple TACE treatments.

In recent years, after sorafenib, more and more targeted drugs, such as lenvatinib, donafenib, and apatinib, are being explored for the adjuvant treatment of HCC after surgery. In addition, numerous studies on the use of single or combined targeted immune checkpoint inhibitors in the adjuvant treatment of HCC after hepatectomy are also being carried out. According to a recent study, patients with China Liver Cancer Staging (CNLC) stage Ⅱ b and/or Ⅲ a are treated with lenvatinib after curative resection, the 1-year RFS rate was 50.5% and the median RFS was 16.5 months^43^. Another study showed that patients with BCLC stage A to B HCC who received donafenib combined with treprinumab after curative resection had an 80% RFS rate at 1-year and a median RFS was not reached^44^. Our result showed that the 1-year RFS rate was 42.0% in LR+TACE group. Although the two studies had a longer RFS than ours, they included more patients with early-stage HCC. Another important reason is that the two studies used combination therapy for postoperative adjuvant treatment, so the efficacy may be better.

Our study has several limitations. First, this study was retrospectively and carried out without randomization, which may have resulted in selection bias, but we tried to minimize such limitation by PSM. Second, since the HCC patients in our study were mainly caused by HBV infection, the results of this study may not be applicable to HCC patients associated with HCV infection or alcohol-related HCC. Third, our study did not address which patients might benefit most from the addition of TACE after LR. Additional studies are needed to reach more reliable conclusions about this issue.

## Conclusion

In conclusion, postoperative adjuvant TACE could be beneficial for intermediate-stage HCC patients with MVI. A single TACE treatment after surgery can maximize the patient’s benefit without the need for multiple TACE treatments. Postoperative TACE should be considered selectively in intermediate-stage HCC patients with MVI after curative LR.

## Ethical approval

This study was conducted in accordance with the Declaration of Helsinki and approved by the Ethics Committee of Hunan Provincial People’s Hospital (approval number: 2022-99), and has been registered in the Chinese clinical trial registry (ChiCTR2300071111).

## Consent

The need for informed consent was waived due to the retrospective nature of the study.

## Sources of funding

This research was funded by Hunan Provincial Natural Science Foundation of China (2023JJ40385).

## Author contribution

C.X., X.S., T.H., and J.Y.: conceptualization and design; C.X., X.S., X.Z., Y.Z., Z.M., and G.Z.: acquisition of data, analysis, and interpretation of data; C.X. and X.Z.: writing – original draft; X.S., T.H., and J.Y.: supervision. All authors contributed in writing – review and editing.

## Conflicts of interest disclosure

The authors declare that they have no conflicts of interest.

## Research registration unique identifying number (UIN)

1. Name of the registry: Chinese Clinical Trial Registry (www.chictr.org.cn).
2. Unique identifying number or registration ID: ChiCTR2300071111.

## Guarantor

Xin Song, Department of Hepatobiliary Surgery, The First Affiliated Hospital of Jishou University, Jishou 416000, Hunan province, People’s Republic of China. E-mail: 13974382020@139.com.

Tao Huang, Department of Minimally Invasive Intervention, Sun Yat-sen University Cancer Center; State Key Laboratory of Oncology in South China, Guangzhou 510060, Guangdong province, People’s Republic of China. E-mail: huangtao@sysucc.org.cn.

Juan Yang, Department II of General Surgery, Hunan Provincial People’s Hospital (The First Affiliated Hospital of Hunan Normal University) Changsha 410005, Hunan province, People’s Republic of China. E-mail: 714514211@qq.com.

## Data availability statement

Please contact the corresponding author by e-mail to obtain data and materials.

## Provenance and peer review

Not commissioned, externally peer-reviewed.
